# Added Value of Antigen ELISA in the Diagnosis of Neurocysticercosis in Resource Poor Settings

**DOI:** 10.1371/journal.pntd.0001851

**Published:** 2012-10-18

**Authors:** Sarah Gabriël, Joachim Blocher, Pierre Dorny, Emmanuel Nji Abatih, Erich Schmutzhard, Michaeli Ombay, Bartholomayo Mathias, Andrea Sylvia Winkler

**Affiliations:** 1 Department of Biomedical Sciences, Institute of Tropical Medicine, Antwerp, Belgium; 2 Department of Neurology, Medical University of Innsbruck, Innsbruck, Austria; 3 Department of Neurology, University Medical Centre Göttingen, Göttingen, Germany; 4 Mental Health Unit, Haydom Lutheran Hospital, Mbulu, Tanzania; 5 Department of Neurology, Technical University Munich, Munich, Germany; Universidad San Francisco de Quito, Ecuador

## Abstract

**Background:**

Neurocysticercosis (NCC) is the most common cause of acquired epilepsy in Taenia solium endemic areas, primarily situated in low-income countries. Diagnosis is largely based upon the “Del Brutto diagnostic criteria” using the definitive/probable/no NCC diagnosis approach. Neuroimaging and specific T. solium cysticercosis antibody detection results are at the mainstay of this diagnosis, while antigen detection in serum has never been included. This study aimed at evaluating the addition of antigen detection as a major diagnostic criterion, especially in areas where neuroimaging is absent.

**Methods:**

The B158/B60 monoclonal antibody-based enzyme-linked immunosorbent assay (ELISA) for the detection of circulating cysticercus antigen was carried out retrospectively on serum samples collected during a hospital-based study from 83 people with epilepsy (PWE) in an endemic area.

**Results:**

The addition of antigen results as a major criterion allowed the correct diagnosis of definitive NCC in 10 out of 17 patients as opposed to 0/17 without antigen results in the absence of neuroimaging. A sensitivity of 100% and a specificity of 84% were determined for the diagnosis of active NCC using antigen ELISA. While the use of a higher cutoff improves the specificity of the test to 96%, it decreases its sensitivity to 83%.

**Conclusions:**

In areas where neuroimaging is absent, NCC diagnosis according to the existing criteria is problematic. Taking into account its limitations for diagnosis of inactive NCC, antigen detection can be of added value for diagnosing NCC in PWE by supporting diagnostic and treatment decisions. Therefore, we recommend a revision of the “Del Brutto diagnostic criteria” for use in resource poor areas and suggest the inclusion of serum antigen detection as a major criterion.

## Author Summary

Neurocysticercosis is a parasitic infection of the central nervous system and a common cause of epilepsy in *Taenia solium* cysticercosis endemic countries. According to the current diagnostic criteria proposed by Del Brutto and colleagues, the diagnosis of neurocysticercosis is mainly based on neuroimaging and detection of specific antibodies. Unfortunately, especially neuroimaging is rarely available in endemic countries. The authors analyzed the value of a test that detects antigens that are excreted by living cysts in people with epilepsy. Different diagnostic scenarios and cut-off values are discussed with the respective sensitivity and specificity of the test. When using the antigen-detecting test, considerably more people with epilepsy were diagnosed correctly with neurocysticercosis. There are some concerns about possible false positive results in other cases. The test was useful for the detection of people with living cysts (active neurocysticercosis), who need further diagnostic evaluation and specific treatment. The authors recommend the addition of this test in the diagnostic criteria for neurocysticercosis.

## Introduction

More than 80% of people with epilepsy (PWE) live in low-income countries [1], where the prevalence of active epilepsy is approximately twice that of high-income countries [2]. Moreover, in many of those countries over 75% of PWE have no access to treatment with anti-epileptic medication [3].

Infectious diseases play a major role in the etiology of epileptic seizures and epilepsy in developing countries [1]. A recent review reported that 29% of PWE also had neurocysticercosis (NCC) [4], caused by the larval stage of *Taenia solium*, a zoonotic parasite.

The treatment of NCC depends on the stage of the disease and the number and localization of lesions. The determination of an optimal treatment is still a developing field of research, it may have to be tailored to individual cases and relies largely on results of neuroimaging techniques. However, there is frequently no or very limited access to/availability of these neuroimaging tools in low-income endemic countries.

To assist in diagnosis, a number of immunodiagnostic tests have been developed, among which is the enzyme-linked immunoelectrotransfer blot (EITB) that detects specific antibodies against *T. solium* cysticerci in serum and was reported to have a high specificity (100%) and sensitivity (98%) [5], [6]. This test is widely recognized; unfortunately it is expensive and in a format (Western Blot) not very applicable in most resource-poor laboratories in endemic areas. More field applicable enzyme-linked immunosorbent assay (ELISA) formats have been developed to detect specific antibodies and antigens in the serum, although they have until now failed to produce consistently good results of high specificity and high sensitivity [6]. However, research is ongoing into the development/identification of new markers for diagnostic tools [7]–[9]. The current antigen detecting ELISA's are based on monoclonal antibodies that detect excretory/secretory proteins produced by viable cysts [10], [11]. As such, these tests detect viable cysts only, which has several epidemiological and clinical implications. In epidemiological studies, the presence of antigens indicates presence of infection, whereas presence of antibodies indicates exposure to the parasite, but not necessarily establishment of infection [12]. For the B158/B60 monoclonal antibody-based antigen ELISA a sensitivity of 90% (95% CI: 80%–99%) and a specificity of 98% (95% CI: 97%–99%) were determined for the detection of infected individuals, based on Bayesian analyses [12].

Currently, the only published diagnostic criteria are the “Del Brutto diagnostic criteria” [13]. However, these criteria have not been systematically validated [14]. Neuroimaging and EITB results provide the basis for most absolute and major criteria, while antigen detection in serum has never been included in the criteria.

The aim of this study was to determine the added value of specific antigen detection in the diagnosis of NCC related epilepsy. Detection of circulating *T. solium* cysticercosis antigen was performed retrospectively on samples from PWE obtained from a hospital-based study carried out in northern Tanzania, in which clinical examinations, CT scanning and antibody detection had been carried out [15]–[17].

## Materials and Methods

### Ethical statement

The study and the use of human subjects for the study were approved by the National Institute for Medical Research (NIMR), Tanzania. The samples were anonymized and transported in accordance to a material transfer agreement between HLH, NIMR, the Medical University Innsbruck, CDC, and ITM.

Written informed consent was obtained from all participants or legal custodians in case of minors. PWE received free treatment for epilepsy and, in case of active NCC, anthelmintic treatment according to national guidelines.

### Recruitment of participants

The study took place at Haydom Lutheran Hospital (HLH) situated in a remote area in the North of Tanzania. The serum and CSF samples were collected during a hospital-based study that has been described elsewhere [15]–[17]. Briefly, 212 PWE, all above 10 years of age, diagnosed by a neurologist (ASW) were followed up and computer tomography (CT) scans were performed. Epilepsy was defined as two or more unprovoked epileptic seizures and categorized according to an International League Against Epilepsy adjusted classification for resource-poor countries [18]. Venous blood samples were taken from 83 of the 212 examined PWE, including 28 of 29 PWE with highly suggestive or definite NCC lesions on CT scan, 7 of 9 PWE with lesions compatible with NCC and 48 of 126 without NCC lesions on CT scan. Due to ethical reasons it was not possible to take blood from all PWE. In addition 11 CSF samples from PWE with multiple cysts or calcifications on CT scan were collected.

### Diagnosis of neurocysticercosis

The diagnosis of NCC was based on the diagnostic criteria proposed by Del Brutto *et al.* (2001) [13]. The combination of absolute, major, minor and epidemiological criteria lead to the diagnoses of definitive, probable or no NCC (Annex S1). All participants had an epidemiological criterion, because Tanzania is endemic for cysticercosis [19]. Epilepsy is a clinical manifestation suggestive of NCC; hence all PWE had one minor criterion. In the present study, CT scans with contrast were available from all PWE. The CT scanner was a Toshiba Auklet Slice Spiral CT. The thickness of slices was 5 mm at skull base and 10 mm above the skull. Cystic lesions showing the scolex were regarded as an absolute criterion, multiple parenchymal calcifications and ring enhancing lesions, which are highly suggestive of NCC, as a major criterion and lesions that are compatible with NCC including equivocal single parenchymal calcifications as a minor criterion (Annex S1). Cystic and ring enhancing lesions were regarded as active NCC and calcifications only as inactive NCC.

### Collection and analysis of serum and cerebrospinal fluid samples

All samples were collected between May and August 2006 at HLH. After clotting, blood samples were centrifuged at 1000×/min for 5 minutes and serum was separated. All samples were initially kept at 4–8°C and after transport in September 2006 at −20°C. The Centers for Disease Control and Prevention, Atlanta, USA (CDC) performed the CDC-developed EITB [5]. Results of the latter analysis were published previously [16].

Samples were analyzed using the B158/B60 antigen ELISA (Ag-ELISA) at the Department of Biomedical Sciences of the Institute of Tropical Medicine, Antwerp, Belgium (ITM) [20]. Eight negative and 2 positive control serum samples were run on each plate. The plates were read using an automated spectrophotometer at 490 nm with a reference of 655 nm. The optical density (OD) of each serum sample was compared with a sample of negative serum samples (n = 8) at a probability level of p = 0.001 (cut-off calculation). A ratio for each sample was calculated by dividing the mean OD of the sample (samples were tested in duplicate) by the cut-off [21]. These ratios were used in the Receiver Operating Characteristic analysis (see data analyses). CSF samples were run using the same methodology and cut-off calculation, albeit the pre-treatment of samples with trichloroacetic acid was not carried out. CSF samples were diluted ½ in phosphate buffered saline.

### Data analysis

The evaluation of the accuracy of Ag-ELISA was performed using a Receiver Operating Characteristic (ROC) analysis. This was done using “probable and definitive NCC” versus “no NCC” and “active” versus “inactive and no NCC” as reference tests (as determined by the “Del Brutto diagnostic criteria”) each in turn. The optimal cut-off ratio value was selected as the point on the ROC curve (which displays estimated percentages of sensitivity and specificity at a selected cut-off value) with the minimum distance to the (0, 1) coordinate. The ROC curves were generated using the R software package [22]. The Fisher's exact test was used to compare circulating antigen levels in different diagnostic groups. Statistical significance was arbitrated at the 5% level. The Mann-Whitney U test was used to compare two groups in a non parametric variable.

## Results

### General aspects

Of the 83 PWE in whom antigen testing was performed, 34 were diagnosed with NCC (following the “Del Brutto diagnostic criteria”), of which 17 were cases of definitive NCC and 17 of probable NCC. Six out of the 17 definitive NCC cases had active NCC lesions on CT scan.

About twenty seven percent (22/83) of PWE were positive on serum Ag-ELISA. In the group of PWE with NCC (according to the “Standard Del Brutto Diagnostic criteria”), circulating antigens were detected in 44.1% (15/34); 58.8% (10/17) in people with definitive NCC and 29.4% (5/17) in people with probable NCC. In the group of PWE without NCC, 14.3% (7/49) were positive on serum Ag-ELISA. The difference in proportion between PWE with NCC and PWE without NCC appeared to be statistically significant (p = 0.005, Fisher's exact test).

**Figure 1:**
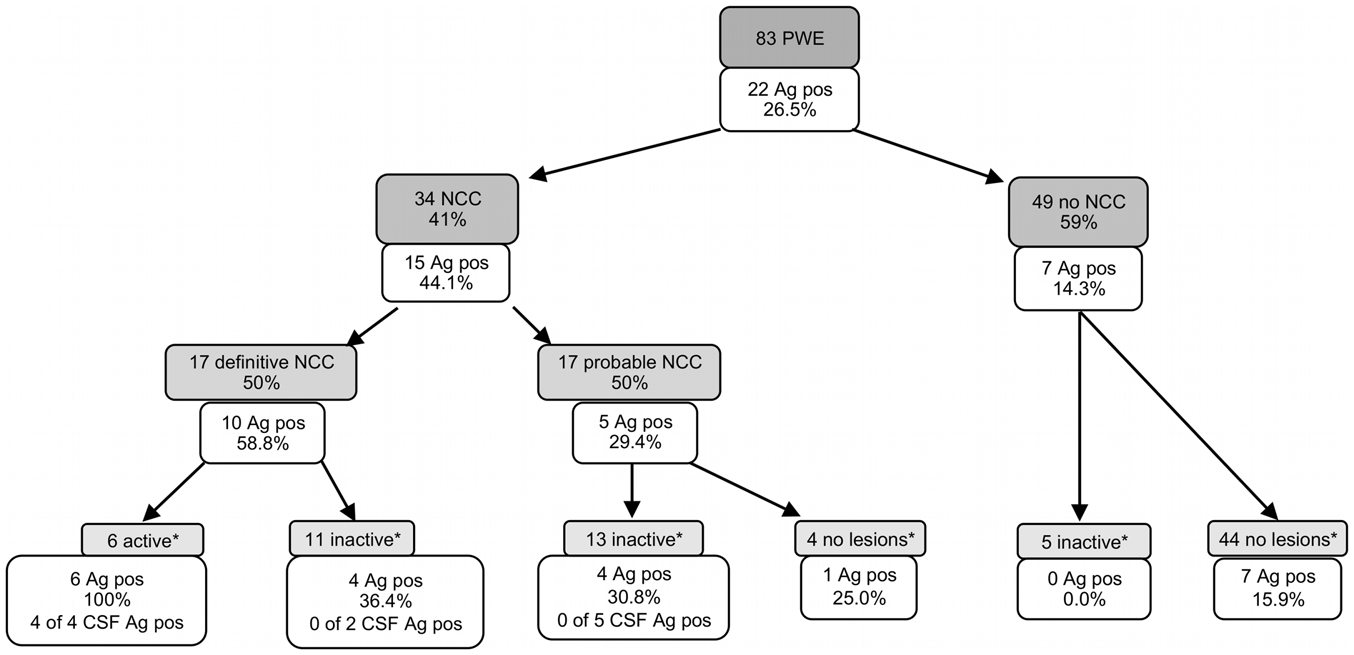
Neurocysticercosis diagnosis according to Del Brutto *et al.* (2001)^22^ and percentage antigen ELISA positivity. Shaded boxes indicate the “Del Brutto diagnoses” (except for *), non-shaded boxes indicate the number and percentage of antigen positive cases. NCC = neurocysticercosis, Ag pos = antigen ELISA positivity, CSF = cerebrospinal fluid, PWE = people with epilepsy. *Active, inactive or no lesions according to CT scan results.

In the group of people with NCC, 100% of active NCC cases (6/6) had a positive Ag-ELISA result in serum, which was significantly higher compared to people with inactive lesions, in whom 33.3% (8/24) were positive on antigen detection (p = 0.005, Fisher's exact test) (Figure 1).

The number of NCC lesions (active or inactive) was significantly associated with a positive Ag-result (p = 0.009, Mann-Whitney U).

Four out of eleven (36.4%) CSF samples were positive on Ag-ELISA. All patients with a positive result based on CSF were also positive based on serum. Regarding diagnosis of NCC, all people with probable NCC were negative on CSF Ag-ELISA (0/5) and 66.7% (4/6) with definitive NCC were positive. Whereas all samples from people with active NCC (4/4) were positive, all samples from people with inactive NCC were negative (0/7).

### Neuroimaging

In Table 1, a comparison is made between different types of NCC lesions and circulating antibody and antigen results in serum and CSF.

**Table 1:** Neurocysticercosis lesions on cerebral CT scan, antigen ELISA and EITB results in serum and cerebrospinal fluid.

		Neuroimaging				EITB (Serum)		EITB (CSF)	
		Absolute NCC	Highly suggestive NCC	Compatible NCC	No NCC	pos	neg	pos	neg
**N° of active NCC**		5	1	0	0				
**Ag-ELISA**	**pos**	5 (100%)	7 (30.4%)	2 (28.6%)	8 (16.7%)	13 (56.5%)	9 (15.5%)	4 (80%)	1 (16.7%)
**(serum)**	**neg**	0 (0%)	16 (69.6%)	5 (71.4%)	40 (83.3%)	10 (43.5%)	49 (84.5%)	1 (20%)	5 (83.3%)
**Ag-ELISA**	**pos**	3 (100%)	1 (12.5%)	n/a	n/a	4 (66.6%)	0 (0%)	4 (80%)	0 (0%)
**(CSF)**	**neg**	0 (0%)	7 (87.5%)	n/a	n/a	2 (33.3%)	5 (100%)	1 (20%)	6 (100%)

NCC = neurocysticercosis,

EITB = enzyme-linked immunoelectrotransfer blot detecting specific antibodies against *T. solium* cysticerci,

Ag-ELISA = B158/B60 monoclonal antibody-based enzyme-linked immunosorbent assay.

CSF = cerebrospinal fluid.

### Del Brutto diagnostic criteria

In Table 2, the “Standard Del Brutto diagnosis” ( = using neuroimaging, EITB, clinical, serological and epidemiological data; annex S1) is compared with the Del Brutto diagnosis without neuroimaging and 1) only EITB is used as a major criterion; 2) only Ag-ELISA is used as a major criterion; 3) EITB and Ag-ELISA are used as major criteria. In the last row, we have determined the change of diagnosis using the “Standard Del Brutto diagnosis” with Ag-ELISA as an added major criterion.

**Table 2:** Comparison of different scenarios of combinations of results from serodiagnosis and neuroimaging.

“Standard Del Brutto” diagnosis		Definite NCC (n = 17)^e^			Probable NCC (n = 17)^f^			No NCC (n = 49)^g^		
New diagnosis of NCC		Definitive NCC	Probable NCC	No NCC	Definitive NCC	Probable NCC	No NCC	Definitive NCC	Probable NCC	No NCC
“Del Brutto diagnosis” without neuro-imaging	EITB only major	0	17	0	0	6	11	0	0	49
	Ag-ELISA only major	0	10	7^a^	0	5	12	0	7^c^	42
	EITB & Ag-ELISA both majors	10^d^	7	0	3^b^	5	9	0	7^c^	42
“Standard Del Brutto diagnosis” with Ag-ELISA added as major		17	0	0	5^b^	12	0	0	7^c^	42

Comparison of different scenarios: the “Standard Del Brutto diagnosis” with availability of neuroimaging is compared with the “Del Brutto diagnosis” without availability of neuroimaging and only EITB as major criterion (first row); only Ag-ELISA as major criterion (second row); EITB and Ag-ELISA as major criteria (third row); and “Standard Del Brutto diagnosis” with neuroimaging and EITB and Ag-ELISA as major criteria (last row).

a,b,c,d : identifiers, see discussion.

e In the three column below all patients with the diagnosis “Definite NCC” according to the “Standard Del Brutto criteria” with all available tests are analyzed.

f In the three column below all patients with the diagnosis “Probable NCC” according to the “Standard Del Brutto” criteria with all available tests are analyzed.

g In the three column below all patients with the diagnosis “No NCC” according to the “Standard Del Brutto criteria” with all available tests are analyzed.

The 83 PWE from our study are according to the “Standard Del Brutto diagnosis”, divided into 17 cases of definitive NCC, 17 of probable and 49 of no NCC. In the absence of neuroimaging, and if only EITB was considered as a major criterion, the 17 cases of definitive NCC could only be diagnosed as probable NCC. If Ag-ELISA was the only major criterion (in absence of EITB results and neuroimaging), 10 cases would be diagnosed as probable NCC and the others as no NCC. If considering both EITB and Ag-ELISA as major criteria, 10 cases would be diagnosed as definitive NCC and 7 as probable NCC.

Of the 17 cases with probable NCC only 6 and 5 cases can be diagnosed with EITB and Ag-ELISA as only majors, respectively. When combining both tests, 3 diagnoses of definitive NCC are made. When all criteria are considered, with inclusion of Ag-ELISA (last row in the Table), 5 cases are allocated a diagnosis of definitive NCC.

In the group of 49 cases with no NCC diagnosis, the addition of Ag-ELISA as a criterion yields 7 probable NCC diagnoses.

### Antigen-ELISA for the detection of neurocysticercosis in people with epilepsy

**Figure 2:**
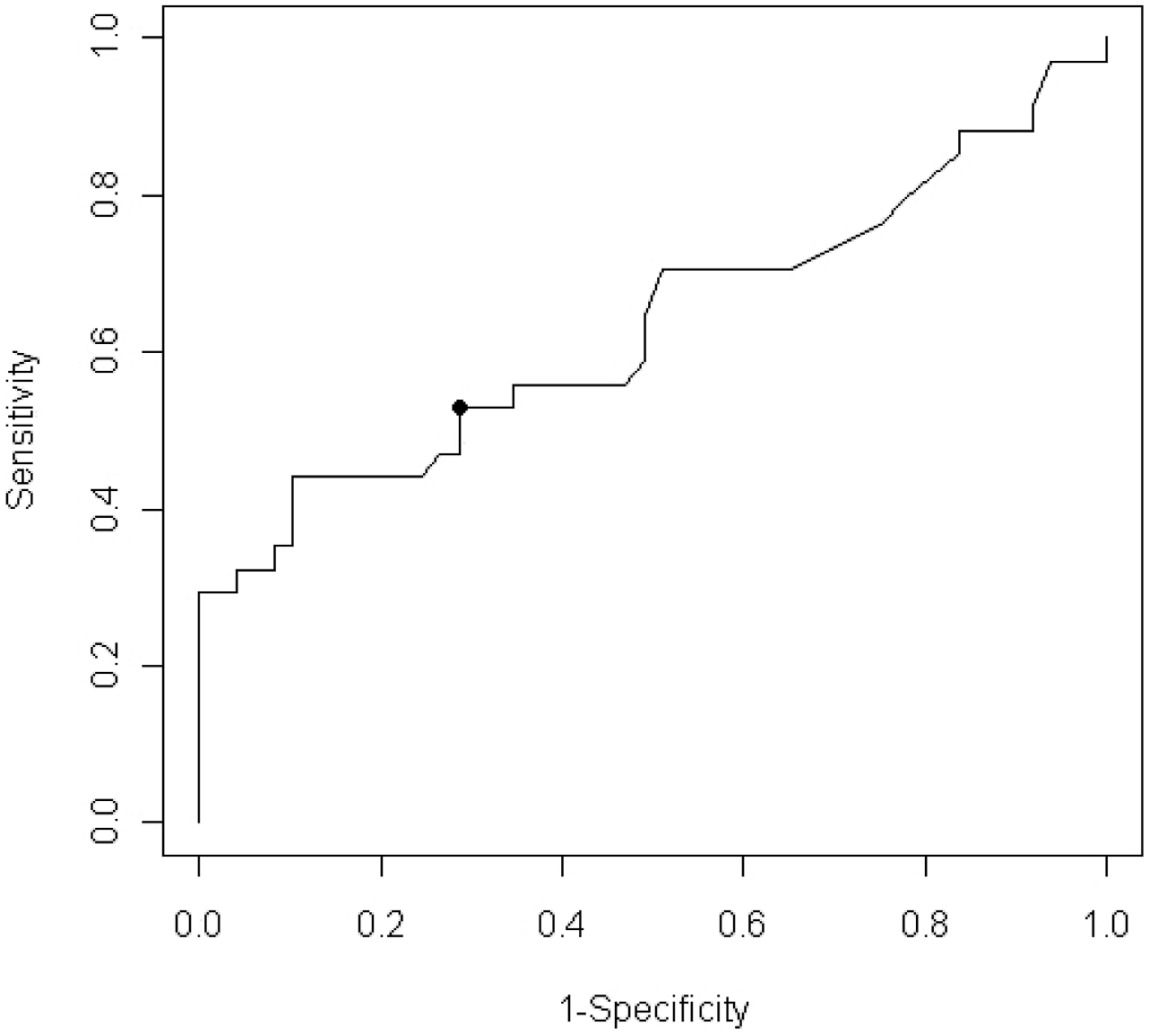
Sensitivity and complement of specificity of antigen ELISA detection of neurocysticercosis in people with epilepsy. Receiver operator characteristic curve showing the relationship between sensitivity (true positive rate) and complement of specificity (false positive rate) for antigen ELISA for the detection of neurocysticercosis in people with epilepsy.

In Figure 2, the ROC curve shows the relationship between sensitivity and the complement of specificity for Ag-ELISA for the detection of NCC in PWE. The dot on the curve indicates the optimal cut-off value corresponding to the maximum sensitivity and specificity (shortest distance to the point (0,1) in the diagram). The optimal cut-off ratio value of 0.81 corresponded to an estimated sensitivity of 53% (95% CI: 37–69%), specificity of 71% (95% CI: 58–82%). The area under the curve was 0.63 (95% CI: 0.52–0.75). If a maximum specificity is requested, an optimal cut-off ratio value of 1.08 can be used, with a sensitivity of 44% (95% CI: 29–61%) and a specificity of 90% (95% CI: 78–96%).

### Antigen-ELISA for the detection of active neurocysticercosis in people with epilepsy

**Figure 3:**
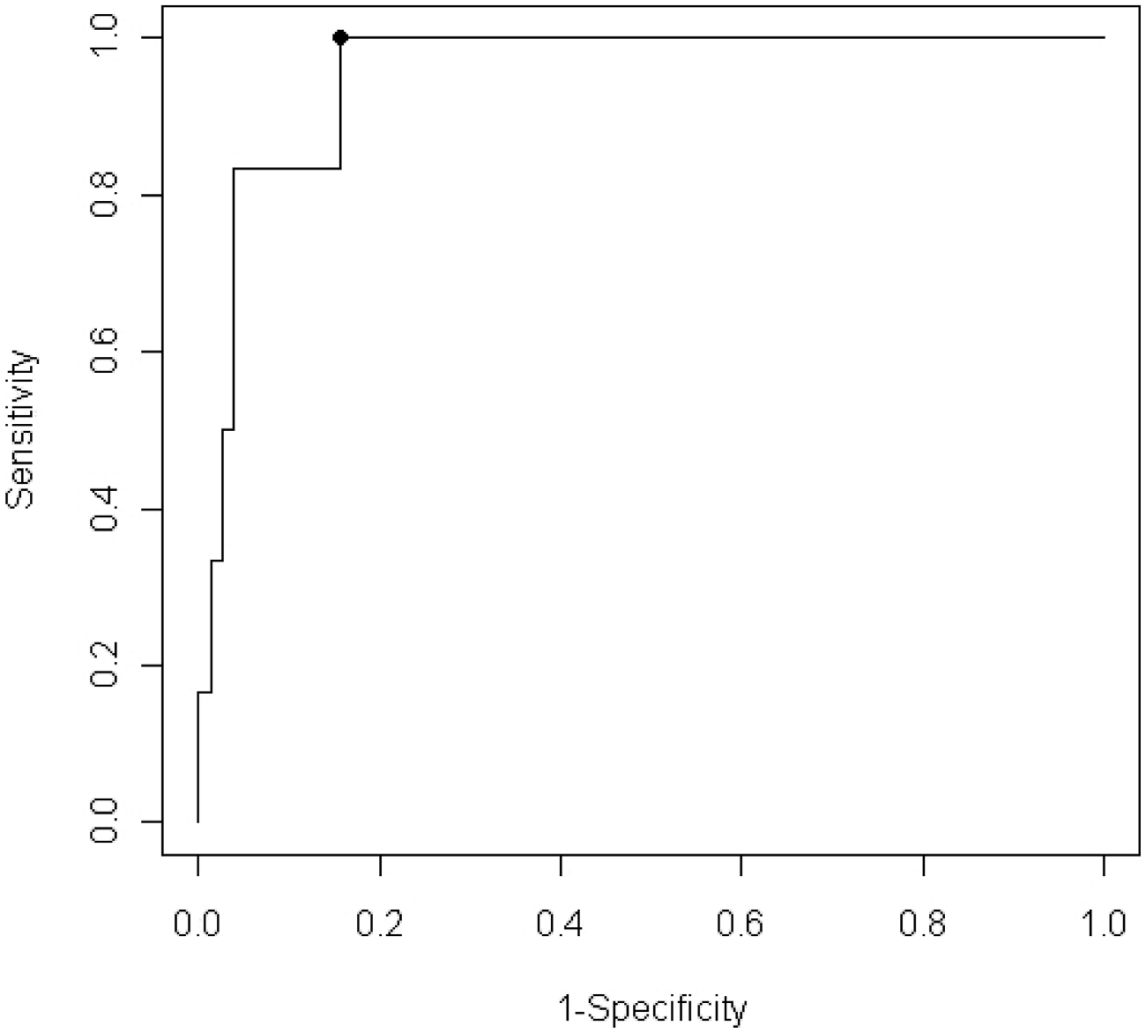
Sensitivity and complement of specificity of antigen ELISA detection of active neurocysticercosis in people with epilepsy. Receiver operator characteristic curve showing the relationship between sensitivity (true positive rate) and complement of specificity (false positive rate) for antigen ELISA for the detection of active neurocysticercosis in people with epilepsy.

In Figure 3, the ROC curve shows the relationship between sensitivity and the complement of specificity for Ag-ELISA for the detection of active NCC in PWE. The optimal cut-off ratio value of 1.17 corresponded to an estimated sensitivity of 100% (95% CI: 61–100%), specificity of 84% (95% CI: 75–91%) and the area under the curve was 0.95 (95% CI: 0.90–1). Considering a prevalence of active NCC of 7.2% (6/83) in our group of PWE, a positive predictive value of 33% and negative predictive value of 100% can be calculated.

If a maximum specificity is requested, an optimal cut-off ratio value of 39.97 can be used, with a sensitivity of 83% (95% CI: 44–97%) and a specificity of 96% (95% CI: 90–99%). At this cut-off ratio, a positive predictive value of 63% and negative predictive value of 99% can be calculated.

## Discussion

The availability of good diagnostic tests for neurocysticercosis (NCC) is essential not only for diagnosis and treatment of individuals, but also for epidemiological studies needed to determine disease burden and evaluation of control programs. In general, diagnosis of NCC is based on the “Standard Del Brutto diagnostic criteria” [13], which are primarily built on neuroimaging. Unfortunately, in most endemic areas, access to neuroimaging is very limited or absent. Therefore, there is an urgent need to assess the validity of these diagnostic criteria in resource-poor areas, and to determine how they can be improved. The aim of this study was to determine the added value of specific antigen detection in the diagnosis of NCC related epilepsy in resource poor settings.

Results indicate a high percentage of antigen positive sera in PWE with active NCC (100% positive), definitive NCC (58.8%) and probable NCC (29.4%), while the percentage is clearly lower in PWE without NCC (14.3%).

These results suggest that circulating antigen detection in serum can be a valuable aid for the diagnosis of NCC. However until now, according to the “Del Brutto diagnostic criteria” [13], antigen detection in serum is not a criterion at all. We have evaluated what effect the addition of serum antigen results as a major criterion would have on NCC diagnosis, in comparison with the “Standard Del Brutto diagnostic criteria”, especially if neuroimaging techniques are not available.

### “Standard Del Brutto diagnostic criteria” and Ag-ELISA

#### Definitive neurocysticercosis

In the absence of neuroimaging, no definitive diagnosis could be made in this study. The use of both EITB and Ag-ELISA as major criteria permits a definitive diagnosis without neuroimaging, as this would yield two major, one minor and one epidemiological criteria according to the “Del Brutto diagnostic criteria” (Annex S1). In our study, this would allow us to diagnose 10 of the 17 patients with definitive NCC (Table 2, d), while with only EITB results at our disposition, we would not be able to determine a definitive diagnosis at all.

When applying only Ag-ELISA results (in the absence of EITB) as major criterion, 7 cases of definitive NCC remain with a no NCC diagnosis (Table 2, a). All these are cases of inactive NCC, which means that no viable cysts were detected on CT scan. This can explain the negative Ag-ELISA result leading to a no NCC diagnosis.

#### Probable and no neurocysticercosis

When considering the Ag-ELISA as a major criterion in the probable NCC diagnosis group, similar results as with the EITB are obtained.

The additional 7 probable NCC diagnoses in the no NCC group (Table 2, c) and additional 3 (5 if neuroimaging is also included) definitive diagnoses in the probable NCC group, all due to Ag-ELISA positivity, could not be confirmed on neuroimaging (Table 2, b). The neuroimaging technique used in this study was CT scan, which is known to have a lower sensitivity for the diagnosis of viable cysts compared to MRI. As such, the presence of viable cysts – as indicated by Ag-ELISA – could have been missed on CT scan [23]–[25]. False positive results due to cross reactions should be considered as well, though no cross reactions were observed when examining sera from patients with parasitologically and/or serologically confirmed infections with *Schistosoma*, hydatid cysts, *Ascaris*, *Trichuris*, filaria, *Entamoeba*, *Plasmodium* and *Trypanosoma* [26]. Alternatively, the Ag-ELISA may cause an overestimation of NCC cases, as positive results may be due to viable cysts lodging elsewhere in the body. The latter is a complicating factor for both serological tests (EITB and Ag-ELISA), as they cannot indicate the position of the cysts in the body. Analyzing CSF could be a helpful tool to localize cysts within the central nervous system. A recent study indicates a significant relationship between antigen levels in CSF and total number of lesions as well as number of non-degenerating cysts as identified by MRI [27]. In our study, all patients with viable cysts on CT scan had a positive CSF Ag-ELISA and all patients with only calcified lesions had a negative result. Therefore CSF analysis might be a useful tool for localizing the infection. However, our sample size was too small to draw a valid conclusion and further studies are needed to confirm these interesting results.

Taking the limitations in the case of inactive NCC into consideration, we strongly believe that the Ag-ELISA has an important added value in NCC diagnosis, especially as it permits a definitive diagnosis based on serological results (EITB and Ag-ELISA), without neuroimaging. Therefore, a revision of the “Del Brutto diagnostic criteria” could be considered towards including the serum Ag-ELISA as a major criterion. The format of the Ag-ELISA is cheap and needs little equipment. It is a monoclonal antibody based test that does not require a continuous supply of fresh parasite material. Moreover, the format has the potential to be adapted into a commercial rapid ELISA kit or lateral flow device. This would make the test more applicable in poorly equipped laboratories with limited financial resources.

### Ag-ELISA and extraparencymal NCC

A less frequent, but nevertheless important manifestation of NCC is extraparenchymal NCC. Due to the design of our study, which included only patients with epilepsy, most of our patients had parenchymal cysts and our results cannot be extrapolated to people with extraparenchymal NCC. However, using the “Del Brutto diagnostic criteria” might lead to an underestimation of *Cysticercus racemosus* forms, because a scolex is not visible [28]. Here also, serum circulating antigen detection could possibly be of great value, as racemose cysts are easily detectable by Ag-ELISA [29].

### Ag-ELISA and implications for clinical diagnosis and treatment

Presence or absence of live cysts can influence treatment decisions as it has consequences on the use of anthelminthics. In the absence (availability and/or access) of neuroimaging, antigen detection could be important to guide further diagnostic and treatment decisions, although treatment with anthelminthic medication based on positive serology results alone clearly is not advisable in resource-poor settings. Furthermore, antigen detection is considered helpful to follow up patients after treatment, where a relatively fast decrease in antigen levels is expected in contrast to antibody levels that can remain positive up to one year after treatment [30].

Clearly the use of the Ag-ELISA to identify NCC cases in a population of PWE (NCC versus no NCC) is less efficient as for the identification of active NCC cases. This can be explained by the number of inactive NCC cases, which the test cannot identify as it detects viable cysts only. However, clinically the differentiation between active NCC and inactive/no NCC is more important than between NCC and no NCC, because the presence or absence of live cysts will influence treatment decisions. Whereas PWE with active NCC should receive antiepileptic drugs, steroids and, depending on number and location of cysts visible on neuroimaging, anthelminthic treatment or even surgery, PWE with inactive or without NCC only need antiepileptic drugs [31]. Positive Ag-ELISA in PWE may help decide in resource-poor settings whether the patient absolutely needs neuroimaging or not, which justifies transportation of the patient to the nearest hospital with neuroimaging facilities.

In conclusion, our results indicate that *T. solium* cysticercosis antigen detection can be of added value for diagnosis of NCC in PWE. As such, besides its use in epidemiological studies [12], the value of antigen results for PWE can be twofold: 1) assist in diagnostic and treatment decisions as it can determine the presence/absence of viable cysts; 2) improve the diagnostic potential, especially in areas where neuroimaging techniques are not available/accessible. It is obvious that Ag-ELISA as a stand alone diagnostic technique cannot be sufficient for the detection of NCC; the use of serological techniques alone is insufficient for this diagnosis [32]. However, clearly more efforts should be put into developing a set of revised diagnostic criteria based on multiple diagnostic tools that can be implemented in resource-poor areas.

## Supporting Information

Annex S1 **Diagnostic criteria according to Del Brutto et al. (2001)^22^.**
(DOC)

Checklist S1 (DOC)

Table S1 **Data of all participants including descriptive diagnosis of imaging, serum and CSF antibody and antigen results and diagnosis of NCC.**
(XLSX)
