# Supplementary material for: Added Value of Antigen ELISA in the Diagnosis of Neurocysticercosis in Resource Poor Settings
Source: PLoS Negl Trop Dis. 2012 Oct 18;6(10):e1851. doi: 10.1371/journal.pntd.0001851 (PMC3475663; doi:10.1371/journal.pntd.0001851)
Supplement: Annex S1 — Diagnostic criteria according to Del Brutto et al. (2001)22. (DOC) [file pntd.0001851.s001.doc]

**Annex 1:** Diagnostic criteria according to Del Brutto *et al.* (2001)^22^
