# Supplementary material for: Added Value of Antigen ELISA in the Diagnosis of Neurocysticercosis in Resource Poor Settings
Source: PLoS Negl Trop Dis. 2012 Oct 18;6(10):e1851. doi: 10.1371/journal.pntd.0001851 (PMC3475663; doi:10.1371/journal.pntd.0001851)
Supplement: Checklist S1 — (DOC) [file pntd.0001851.s002.doc]

# STARD checklist for reporting of studies of diagnostic accuracy

*(version January 2003)*

*GENERAL REMARKS: The presents study is not a study that compares two tests (gold standard) to a new test, hence some points of the STARD checklist are not applicable. The authors evaluated an existing test in a diagnostic setting. However, we tried to stick to the STARD recommendations as close as possible.*

| **Section and Topic** | **Item**  **#** |  | **On page #** |
| --- | --- | --- | --- |
| TITLE/ABSTRACT/  KEYWORDS | 1 | Identify the article as a study of diagnostic accuracy (recommend MeSH heading 'sensitivity and specificity'). | 1 |
| INTRODUCTION | 2 | State the research questions or study aims, such as estimating diagnostic accuracy or comparing accuracy between tests or across participant groups. | 6 |
| METHODS |  |  |  |
| *Participants* | 3 | The study population: The inclusion and exclusion criteria, setting and locations where data were collected. | 7 |
|  | 4 | Participant recruitment: Was recruitment based on presenting symptoms, results from previous tests, or the fact that the participants had received the index tests or the reference standard?  *ANSWER: Recruitment was based on presenting symptoms (epilepsy) and result of CCT scan. For details see page 7.* | 7 |
|  | 5 | Participant sampling: Was the study population a consecutive series of participants defined by the selection criteria in item 3 and 4? If not, specify how participants were further selected.  *ANSWER: The study population consisted of consecutive patients with epilepsy and signs of NCC in cerebral CT scan and controls of people with epilepsy without signs of NCC on CT scan. Not all participants agreed to the blood test and had to be excluded.* | 7 |
|  | 6 | Data collection: Was data collection planned before the index test and reference standard were performed (prospective study) or after (retrospective study)?  *ANSWER: This is a retrospective study. The antigen-ELISA was not part of the initial study design. All available samples were analyzed after the recruitment was closed.* | 7 |
| *Test methods* | 7 | The reference standard and its rationale. | 8 |
|  | 8 | Technical specifications of material and methods involved including how and when measurements were taken, and/or cite references for index tests and reference standard. | 8,9 |
|  | 9 | Definition of and rationale for the units, cut-offs and/or categories of the results of the index tests and the reference standard. | 8,9 |
|  | 10 | The number, training and expertise of the persons executing and reading the index tests and the reference standard. | 8 |
|  | 11 | Whether or not the readers of the index tests and reference standard were blind (masked) to the results of the other test and describe any other clinical information available to the readers.  *ANSWER: The reviewer of the CT scan had no clinical information apart that all patients had epilepsy. Both labs (CDC and ITM) analyzed all samples (serum and CSF) blinded. They had no information about CT result or clinical findings or the result of the other lab.* | 8,9 |
| *Statistical methods* | 12 | Methods for calculating or comparing measures of diagnostic accuracy, and the statistical methods used to quantify uncertainty (e.g. 95% confidence intervals). | 9 |
|  | 13 | Methods for calculating test reproducibility, if done.  *ANSWER: not applicable.* | NA |
| RESULTS |  |  |  |
| *Participants* | 14 | When study was performed, including beginning and end dates of recruitment. | 8 |
|  | 15 | Clinical and demographic characteristics of the study population (at least information on age, gender, spectrum of presenting symptoms).  *ANSWER: Demographic data was published elsewhere (reference 17+18), individual data is presented in supplement table 1.* | Suppl. Table 1 |
|  | 16 | The number of participants satisfying the criteria for inclusion who did or did not undergo the index tests and/or the reference standard; describe why participants failed to undergo either test (a flow diagram is strongly recommended). | 7 |
| *Test results* | 17 | Time-interval between the index tests and the reference standard, and any treatment administered in between.  *ANSWER: not applicable.* | NA |
|  | 18 | Distribution of severity of disease (define criteria) in those with the target condition; other diagnoses in participants without the target condition.  *ANSWER: not applicable.* | NA |
|  | 19 | A cross tabulation of the results of the index tests (including indeterminate and missing results) by the results of the reference standard; for continuous results, the distribution of the test results by the results of the reference standard. | 10-13, Table 1+2, Figure 2+3 |
|  | 20 | Any adverse events from performing the index tests or the reference standard.  ANSWER: The samples were analyzed retrospectively after the recruitment was finished. | NA |
| *Estimates* | 21 | Estimates of diagnostic accuracy and measures of statistical uncertainty (e.g. 95% confidence intervals). | *For antigen ELISA published elsewhere (reference 20)* |
|  | 22 | How indeterminate results, missing data and outliers of the index tests were handled.  *ANSWER: not applicable.* | NA |
|  | 23 | Estimates of variability of diagnostic accuracy between subgroups of participants, readers or centers, if done.  ANSWER: Discussed in detail in the discussion part and table 2 | 14-16  Table 2 |
|  | 24 | Estimates of test reproducibility, if done.  *ANSWER: not applicable.* | NA |
| DISCUSSION | 25 | Discuss the clinical applicability of the study findings. | 14-18 |
